# Parental-caregiver perceptions of child oral health-related quality of life (P-CPQ): Psychometric properties for the peruvian spanish language

**DOI:** 10.4317/medoral.19195

**Published:** 2013-10-13

**Authors:** Ursula Albites, Jenny Abanto, Marcelo Bönecker, Saul M. Paiva, Denisse Aguilar-Gálvez, Jorge L. Castillo

**Affiliations:** 1 DDS, Pediatric Dentistry Department, Dental School, Científica del Sur University - UCSUR, Peru; 2 DDS, MSc, PhD, Pediatric Dentistry and Orthodontics Department, Dental School, University of São Paulo-USP, Brazil; 3 DDS, MSc, PhD, Chairman Professor of Pediatric Dentistry and Orthodontics Department, Dental School, University of São Paulo-USP, Brazil; 4 DDS, MSc, PhD, Chairman Professor of Pediatric Dentistry and Orthodontics, Dental School, Federal University of Minas Gerais -UFMG, Brazil; 5 DDS, MSc, PhD, Chairman Professor of Pediatric Dentistry Department, Dental School, Científica del Sur University - UCSUR, Peru; 6 DDS, MSc, PhD, Professor of Pediatric Dentistry Department, Dental School, Cayetano Heredia University - UPCH, Peru

## Abstract

Objectives: The aim of the study was to cross-culturally adapt the Parental-Caregiver Perceptions Questionnaire (P-CPQ) to the Peruvian Spanish language and assess its reliability and validity. Study Design: To translate and cross-cultural adapt the instrument, 60 parents answered the P-CPQ in two pilot tests. The final version of the P-CPQ was evaluated in 200 parents of children aged 11 to 14 years, who were clinically examined for dental caries. The internal consistency was assessed by Cronbach’s alpha coefficient while repeat administration of the P-CPQ on the same 200 children facilitated the test-retest reliability via intraclass correlation coefficient (ICC). Construct and discriminant validity were based on associations of the P-CPQ with global ratings of oral health and clinical groups, respectively. Results: The mean (standard deviation) P-CPQ score was 15.64 (11.89). Internal consistency was confirmed by a Cronbach’s alpha of 0.84. Test-retest reliability revealed excellent reproducibility (ICC= 0.94). Construct validity was satisfactory, demonstrating significant correlations between global ratings (oral health and overall well-being) and the total scale and for subscale. Discriminant validity was significant (*p*<0.001), supporting its ability to discriminate between clinical groups. Conclusions: The Peruvian Spanish P-CPQ has satisfactory psychometric properties to assess parental-caregivers perceptions on their children’s oral health-related quality of life.

** Key words:**Quality of life, oral health, children, validity, reliability.

## Introduction

The concept of oral health-related quality of life (OHRQoL) relates to the impact that oral conditions have on the individual’s daily functioning, well-being or quality of life (1,2). Oral health is part of overall health and is essential to the quality of life, but this is affected by diseases such as dental caries, which afflicts much of world population, including children (3-7). The children’s health needs are reported by their parents or ca-regivers, and the decisions about them including choice of treatment to be performed are taken according to the perception of parents or caregivers. Based on this, it is recommended that OHRQoL measures should assess parent and child perspectives, in order to obtain additional information (7-10).

The Parental-Caregiver Perceptions Questionnaire (P-CPQ) is one of the instruments of the Child Oral Health Quality of Life Questionnaire (COHQOL), this instrument was developed in English language (9) and has been validated for the English language of Canada, United Kingdom and New Zealand also in Brazilian Portuguese and in Chinese (9-13). The lack of such instruments in Spanish limits it use for oral health research in Spanish-speaking countries, such as Peru. There are three reasons for preferring the cross-cultural adaptation: the complexity of creating a new instrument, allows reliability and validity similar to the original and to standardize studies and comparisons between diffe-rent cultural groups (14-17).

Therefore, the aim of the present study was to carry out the cross-cultural adaptation of the P-CPQ to the Peruvian Spanish language and to test its reliability and validity.

## Material and Methods

-Description of the Parental-Caregiver Perceptions Questionnaire (P-CPQ)

The P-CPQ has 31 items divided in 4 subscales: oral symptoms (6 items), functional limitations (8 items), emotional well-being (7 items), and social well-being (10 items). The questions referred to the frequency of events in the previous 3 months. A five-point Likert scale was used with the following response options: “never”=0, “once/twice”=1, “sometimes”=2, “often”=3, “every day/almost every day”=4.

The P-CPQ scores are calculated as a simple sum of the response codes. Since there were 31 questions, the final score can vary from 0 to 124, for which a higher score denotes a greater degree of the impact of oral conditions on the quality of life of the child.

The authors also designed two questions asking the parents for a global rating of their children’s oral health and the extent to which the oral health affected their overall well-being (9). These global ratings had a five-point response format. The responses were scored as follows: “excellent”=0, “very good”=1, “good”=2, “regular”=3, “poor”=4 for oral health and not at all=0, very little=1, somewhat=2, a lot=3 and very much=4 for general well-being.

-Translation and Adaptation of the P-CPQ 

The PCPQ was translated and adapted to Spanish for Peru according to international guidelines (17-19). Two initial translations were made independently by two bilingual translators, Peruvian fluent in the English language, with experience in the OHRQoL field. Both translations were reviewed in a consensus meeting in Peru. The Revision Panel for this meeting consisted of four postgraduate professors, all fluent in both Spanish and English, who knew the objectives of the study and had experience in OHRQoL studies. This version was compared to the original with special attention to the meaning of the words in the different languages in order conceptual equivalence in the different cultures. An effort was made to identify possible difficulties in understanding the items of the instrument. A consensus forward-translated version was developed as a result of this process. This version was then pilot-tested on a convenience sample of 40 parents/caregivers of children aged 11-14 years old. Parents suggested the substitution of some words or expressions for synonymous to facilitate de comprehension of the questionnaire; modifications were made according the parents comments. The Review Panel reviewed the results and in consensus developed the first Peruvian Spanish version of the PCPQ.

This first version was independently back-translated into English by two translators, whose native language were English and spoke fluent Spanish, they were not previously involve in the study. These two back-translated versions proved nearly identical. The Review Panel compiled in consensus a single version of these translations. To determine semantic equivalence, a group of three dental surgeons fluent in both languages and without previous knowledge of the study compared these back-translated versions with the original one in English, in order to get a “similar effect” on respondents who speaks both languages (English and Spanish).

The first Peruvian Spanish version of the P-CPQ required a second pilot test, which assessed its clarity, appropriateness and cultural relevance (functional equivalence). It was tested on a convenience sample of 20 parents/caregivers, different from the first pilot. There were no changes, new suggestions or difficulties in understanding. This second pilot testing verified the appropriateness and cultural relevance of the target language version. Finally, the Revision Panel formulated the final Peruvian version as a result of this process (Additional File 1).

-Assessment of validity and reliability 

The Peruvian version of the P-CPQ was administered by one interviewer in face-to-face independent interviews to 200 parents/caregivers of children between 11 to 14 years old from four schools, two of them were public schools in a deprived area and the others private schools in a privileged area. All schools were located in the city of Lima, capital of Peru. In order to assess test-retest reliability, the 200 parent/caregivers completed the P-CPQ for a second time, 1 to 2 weeks after the first interview. Children were initially randomly selected from official school registries. All parents were invited to participate in the study according to the following inclusion criteria: parents who have children with no systemic and ⁄ or neurological diseases. Who were able to be examined intra-orally, and with parents who were fluent in Spanish. To avoid possible biases, relatives and children living in the same household were excluded from the study. The study was approved by the Human Research Ethics Committee of the University of São Paulo.

The children’s oral examinations referred to dental ca-ries assessment according to Knutson criteria (20). To assess discriminant validity, children were divided into two clinical groups: those with no dental caries experience (DMFT=0) vs. those with dental caries experience in one or more teeth (DMFT ≥1) (20). The children’s oral examinations were carried out by a single specialist in pediatric dentistry who was previously trained and calibrated (Kappa intra-agreement= 0.92).

-Data analysis

The SPSS software program (version 17.0 SPSS Inc., Chicago, IL, USA) was used for data analysis. Initially descriptive analyses were performed to assess the prevalence of oral impacts and measures of central tendency (means and standard deviations) of total and individual subscale scores of the Peruvian P-CPQ.

The internal consistency of the P-CPQ was assessed using Cronbach’s alpha, inter-item and item-total correlation coefficients (n=200). The reliability was verified by calculating the intraclass correlations coefficient (CCI) with a two way random-effect model, for the P-CPQ score using the data from the same 200 children who were interviewed a second time by the same interviewer.

To test the construct validity, correlations between the scores of each subscale, total score and global ratings were analyzed using Spearman’s correlation coefficient. Discriminant validity was tested by comparing the mean P-CPQ scores between children with caries experience and those without. As the scores P-CPQ scores were not normally distributed, the nonparametric Mann-Whitney test was used to evaluate the difference in mean scores between the two groups. The level of significance was set at 0.05.

## Results

A total of 243 parents were invited to participate in the validation study. Of them, 43 were not included because they did not fulfill the inclusion criteria and of the 200 that were eligible, 200 provided signed parental informed consent, resulting in a response rate of 82.3%.

Of the 200 parents interviewed, 85.0% were mothers and 15.0% fathers. The mean (standard deviation) age of children was 12.5 (1.12), of whom 95 (47.5%) were from public schools and 105 (52.5%) from private schools. Of them 54.0% were girls and 46.0% boys, and a total of 92 children (46.0%) had no caries experience (DMFT= 0) and 108 (54.0%) had experience of dental caries (DMFT≥ 1). All questionnaires were fully completed. The scores for the total scale in the study population ranged from 0 to 57, with a mean (standard deviation) of 15.64 (11.89). Overall, 98.0% of parents reported oral impact (P-CPQ scores >0, considering the “never threshold”). Of them, 180 parents (90.0%) reported experiencing oral symptoms in the previous 3 months; 184 (92.0%) reported functional limitations; 142 (71.0%) reported emotional well-being impacts and 149 (74.5%) reported social well-being impacts.

-Reliability 

Cronbach’s alpha for the total score was 0.84 indicating an excellent internal consistency. Values varied from 0.53 for oral symptoms to 0.73 for social wellbeing subscales. The test-retest reliability was assessed using el ICC, which was 0.94 for the total score and ranging from 0.89 for social well-being to 0.95 for emotional well-being subscales ( Table 1).

**Table 1:**
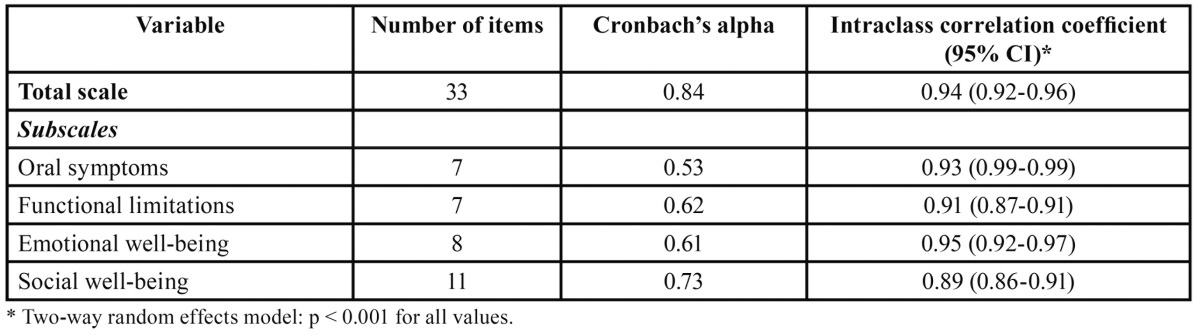
P-CPQ Reliability statistics for total score and subscales (n = 200).

-Construct validity

The correlations between the global ratings (oral health and overall well-being) and the total scale (r= 0.245 and r= 0.286), oral symptoms subscale (r= 0.249 and r= 0.231), functional limitations subscale (r= 0.241 and r= 0.235), emotional well-being subscale (r= 0.237 and 0.198) and social well-being subscale (r= 0.260 and 0.309), were mediocre but statistically highly significant ( Table 2).

**Table 2:**
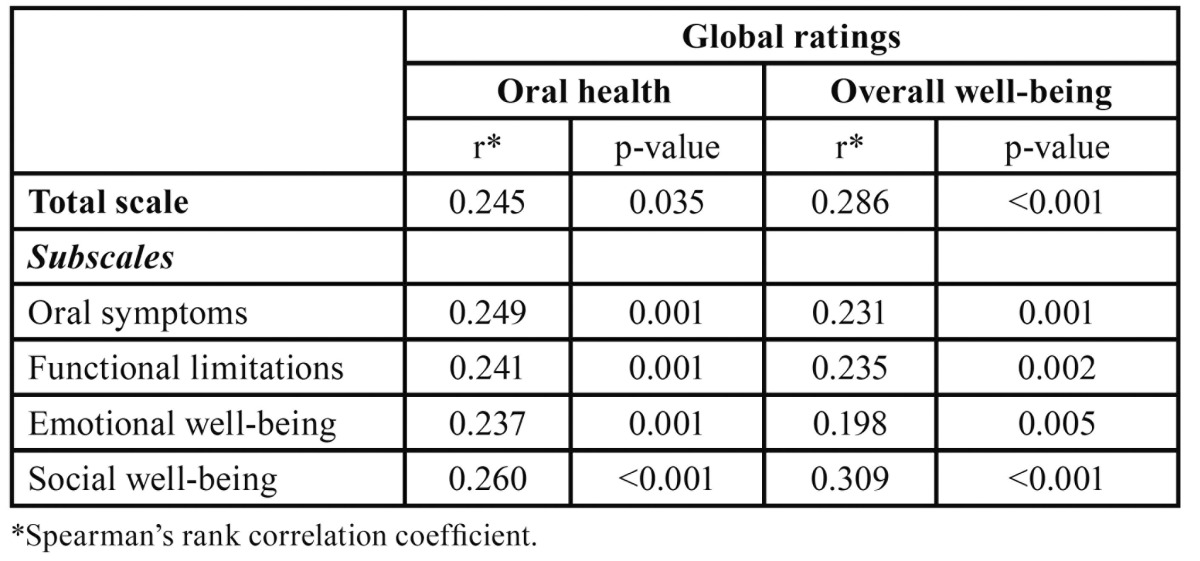
P-CPQ Construct validity: rank correlations between total scale and subscale scores, and global rating of oral health and overall well-being (n = 200).

-Discriminant validity

There was a significant difference in total and subscale scores of the P-CPQ (*p*<0.001) between children without dental caries experience and those with dental ca-ries experience in one or more teeth ( Table 3).

**Table 3:**
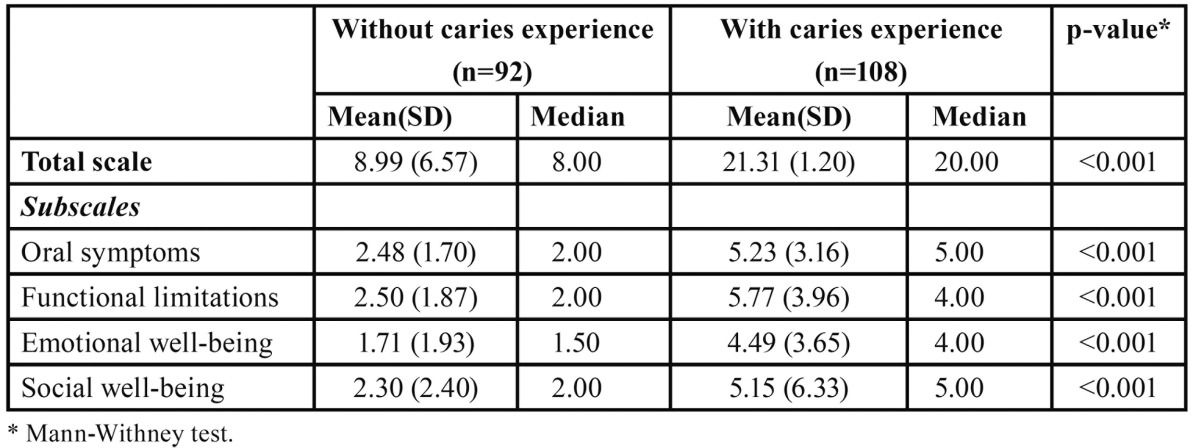
Discriminant validity: overall and subscales scores for children without dental caries experience and those with dental caries experience.

## Discussion

Since long ago, there have been studies that assess the impact of oral disorders on quality of life of individuals. Several instruments serve to this purpose; one of them is the P-CPQ questionnaire, which was originally created for use in the English language. At present, the cross-cultural adaptation and validation of the PCPQ has been done in two other languages: Chinese and Brazilian Portuguese.

In order to use the P-CPQ in a different context and country population, the cross-cultural adaptation to the new language should be evaluated because it allows that the validity and reliability of a measure be similar to the original. Standard guidelines (17-19) describe the initial part of the process applying meticulous translation and back-translation by different qualified translators, the presence of a review panel composed by experts and a pre-test phase that included cognitive interviews to show whether items of the instrument were comprehensible and acceptable. Although this process requires time consuming, it is important because there are points of considerable importance, as is finding the right words into Spanish; including Peruvian’s mind (it is known that there are different expressions in each Spanish-speaking country).

The Peruvian Spanish version of the P-CPQ showed satisfactory validity and reliability, thus indicating its use for assessing children’s OHRQoL according to parent’s perceptions in Peru. Test-retest reliability demonstrated excellent correlations between responses in the first and second time interviews for the whole instrument. Cronbach’s alpha coefficient was 0.84 for the total scale and ranging from 0.53 to 0.73 for subscales, indicating acceptable internal reliability, as values of 0.5 or above are considered acceptable (21). Similar findings were found in the English versions (9,10) and in the Chinese and Brazilian Portuguese versions (10,12,13), however, while oral symptoms subscale presented a moderate internal consistency in this study, this value differ slightly from those reported by other authors, being higher values for the English and the Chinese versions (9,13).

Concerning construct validity, significant correlations were found in this study between global ratings and the total scale and subscales. The other versions of the P-CPQ found similar results except in the correlation of oral symptoms subscale with overall well-being rating (9,10,13). As we expected, the mean total scores and for subscales were statistically higher in the dental caries experience group indicating the ability of the Peruvian P-CPQ to significantly discriminate between different clinical groups. Comparisons about discriminant validity cannot be done with others version of the P-CPQ due to the use of different indices for evaluating and analyzing caries condition.

## Conclusion

The Peruvian Spanish version of the P-CPQ exhibited satisfactory psychometric properties regarding its validity and reliability and can be used to assess parental-caregi-vers perceptions on their children’s OHRQoL in Peru.
